# Erythema Multiforme Attributable to Herpes Simplex Virus: Clinical Aspects and Treatment

**DOI:** 10.1155/2021/6692495

**Published:** 2021-05-10

**Authors:** Aya Mtiri, Ghada Bouslama, Nour-sayda Ben Messouad, Iyadh Abidi, Souha Ben Youssef, Badreddine Sriha

**Affiliations:** ^1^Department of Oral Surgery, University Hospital Farhat Hached Sousse, University of Monastir, Tunisia; ^2^Department of Histopathology, University Hospital Farhat Hached, Sousse, Tunisia

## Abstract

Erythema multiforme is an acute mucocutaneous hypersensitivity reaction with various etiological factors, including herpes simplex virus, medications, autoimmune diseases, and malignancies, but the most common cause is infection by herpes simplex virus. The most characteristic feature is the presence of “target lesions.” There are no specific diagnostic tests for EM, and the diagnosis is based on clinical signs and symptoms and biopsy if required. We report a case of recurrent herpes-associated erythema multiforme managed with prophylactic acyclovir for 6 months: a 17-year-old boy had recurrent cutaneous lesions as well as lesions in the oral cavity and lips. Positive serology for herpes simplex virus and histopathological examination confirmed the diagnosis.

## 1. Introduction

Erythema multiforme (EM) is a reactive mucocutaneous disorder that is characterized by acute cutaneous and mucous bullous lesions. It usually affects adolescents and apparently healthy young adults but may occur at any age, and several reports suggest that males are affected more than females [1].

The detachment of the skin affects less than 10% of the body surface area (BSA), and localized typical and/or raised atypical targets are present.

## 2. Case Report

A 17-year-old male patient was referred from the internal medicine department to the dental surgery unit of University Hospital Farhat Hached, Tunisia. The patient was complaining about recurrent intraoral ulcers, diagnosed with recurrent aphthous stomatitis. He was treated with colchicine for two months without any improvement.

Our patient was afebrile with all vital signs within normal limits, and his medical history was noncontributory.

**Figure 1:**
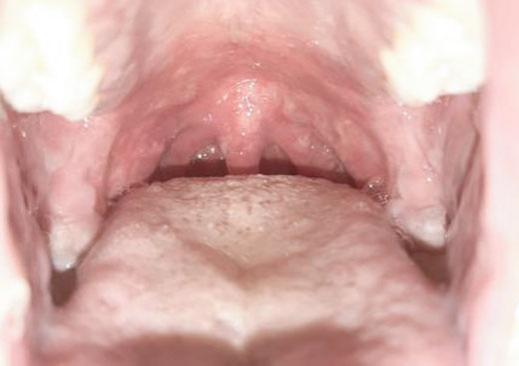
Mucosal involvement of the palate.

Extraoral examination at this point revealed nonspecific 3 to 4 purplish red cutaneous lesions on the forearms. Only some itching was reported. However, intraoral examination revealed diffuse and painful irregular erythematous lesions and postbullous ulcerations on the palate (Figure 1) and all buccal mucosae. Moreover, poor oral hygiene was noticed as the patient was unable to brush his teeth regularly.

Based on the clinical examination, the diagnosis of recurrent aphthous stomatitis was excluded. Yet a bullous disease was suspected such as bullous pemphigoid, lichen planus, or even a drug reaction related to the use of colchicine. Aimed at confirming our diagnosis, a histological exam with a biopsy in the left buccal mucosa was performed.

The patient was told to stop all medications and start using corticosteroids: Solupred (20 mg) 1 tablet ∗3 per day for 20 days and a topical steroid for the cutaneous lesions in order to relieve him and enable him to eat and speak conveniently.

**Figure 2:**
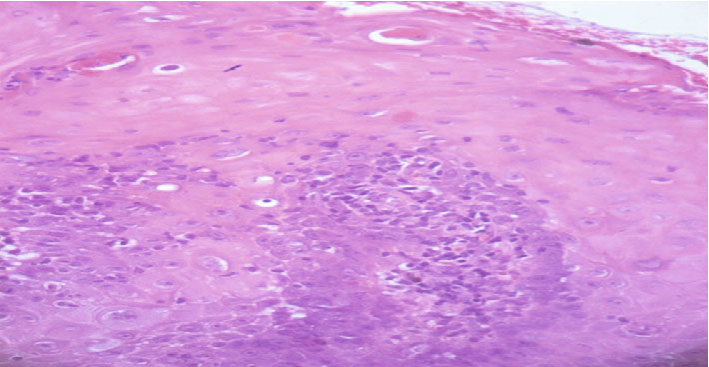
Histopathology of EM minor. A dermal inflammatory infiltrate consisting of lymphocytes, histiocytes, and necrotic epidermal cells.

One week later, the patient showed a clear improvement of mucosal lesions as well as pain relief. The histopathological analysis showed a prominent dermal inflammatory reaction with vasodilation and oedema. A dermal inflammatory infiltrate consisting of lymphocytes and histiocytes was identified as well as some necrotic epidermal cells (Figure 2). Thus, the diagnosis of erythema multiforme was retained.

**Figure 3:**
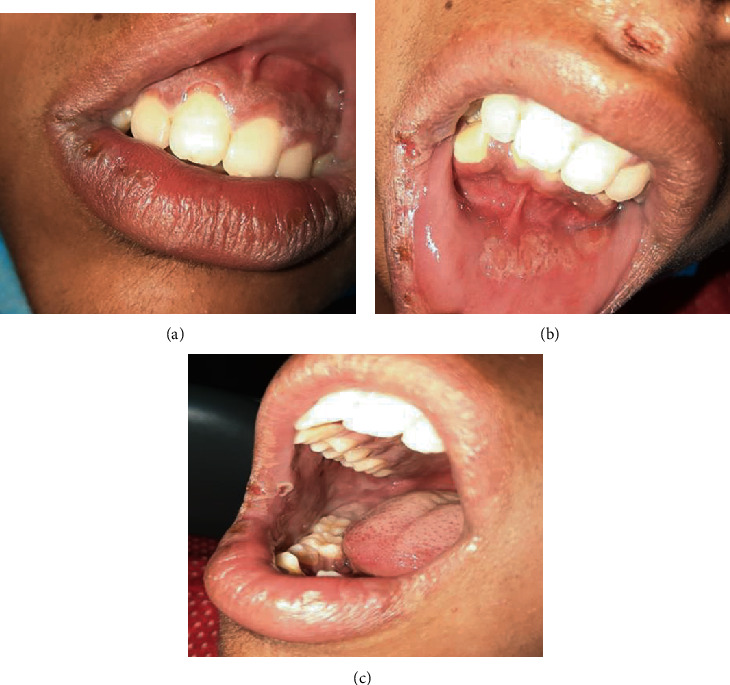
Oral lesions of oral erythema multiforme. The oral lesions involve (a) the gingival, (b) labial, and (c) buccal mucosae and vermilion of the lips.

Unfortunately, one month later, the patient reported complaints about more severe postbullous lesions on buccal, gingival, and labial mucosae (Figure 3). The lesions were diffuse, multiple, irregularly mixed, red and white, surrounded by erythematous margins, and covered with white slough in addition to postvesicular heme-crusted polycyclic erosions of vermillion lips and philtrum.

**Figure 4:**
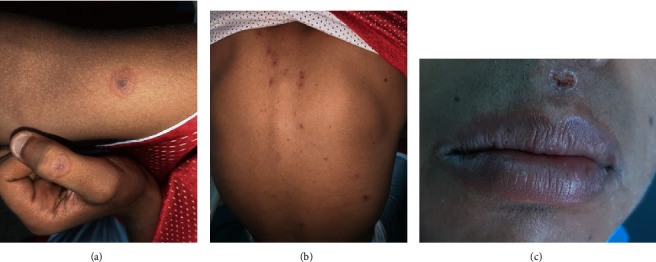
Clinical presentation of EM: (a) target lesions on the right arm and left thumb; (b) target lesions on the back; (c) crusty blister.

Furthermore, numerous targetoid erythematous lesions over the back of hands, fingers, arms, and back were noticed (Figure 4).

Considering the presence of a herpes lesion and the recurrence in a short period of time, a correlation between herpes simplex infection and erythema multiforme was suspected. Therefore, serology tests were indicated. The patient was negative for human immunodeficiency virus (HIV) while positive for herpes simplex virus (HSV) pinpointing a chronic infection (IgM: negative; IgG: positive).

Clinical evidences, positive serology tests, and pathological findings were in accordance with diagnostic features of HAEM.

The patient was treated with prednisolone 60 mg a day for 10 days, antiseptic, analgesic, and anesthetic mouthwash, topical steroid ointment, and valaciclovir 500 mg a day for 6 months to prevent the recurrence.

The patient when recalled after 2 months showed marked improvement in his condition with no signs of recurrence.

## 3. Discussion

The diagnosis of EM is based on the clinical appearance supported if necessary by biopsy.

EM patterns can be classified into EM with and without mucosal involvement: the oral cavity and the genital, ocular, laryngeal, and esophageal mucosae may be affected.

EM has been subdivided by some authors [2] into EM minus (involvement of ≤1 mucosal site) and EM majus (involvement of ≥2 mucosal sites).

Typical targets are defined as lesions less than 3 cm in diameter and characterized by three different concentric zones with predominant acral localization usually symmetrical. Raised atypical targets may occur containing only two zones. In typical and raised atypical targets, the center zone might show bulla formation as a sign of epidermal involvement [2, 3].

Oral involvement may precede lesions on other sites or may arise in isolation [1]. It is usually presented by lesions progressing through diffuse macules to blisters and ulceration, lips becoming swollen, bleeding, and crusted and intraoral lesions typically on the nonkeratinized mucosae and most pronounced in the anterior parts of the mouth.

Infections are the most common cause. Herpes simplex virus (HSV) infection is the most commonly identified cause of EM. Assier et al. identified HSV infection in 17 of 28 patients with recurrent EM. 23% of patients were found to have associated HSV infection [2, 4]. EM typically follows a lesion of recurrent herpes simplex within 1 to 3 weeks. The interval usually is about 10 days; one study found that the lip was the most common site of preceding HSV infection in recurrent EM implicating HSV-1 [1] just like our case. Nonetheless, many causative factors have been implicated including bacterial, viral, and fungal infections, drugs, radiation therapy, and emotional stress [5].

A characteristic histopathological finding of EM is necrosis of some keratinocytes and epidermal damage in the form of basal cells. This is particularly notable in the center of the target lesions of EM [5, 6]. In more severe bullous cases, necrosis of the whole dermis is noted [7, 8].

Direct immunofluorescence is performed to confirm the diagnosis as well as to rule out other diseases with diagnostic immunofluorescence findings such as autoimmune mucocutaneous diseases, in particular pemphigus vulgaris and paraneoplastic pemphigus, mucosal bullous pemphigoid, and linear IgA dermatosis.

There are significant differences in severity and clinical expression between EM and Stevens-Johnson syndrome (SJS) that cause widespread lesions and epithelial detachment involving less than 10% of the body surface and toxic epidermal necrolysis (Lyell's disease, TEN) characterized by extensive detachment of the full-thickness epithelium usually induced by drugs [1, 9]. During clinical investigations, primary herpetic infection, hand-foot-and-mouth disease, urticaria, lupus erythematosus, erosive lichen planus, fixed drug eruption, cutaneous vasculitis, and some neutrophilic dermatoses have to be considered in the differential diagnosis of EM [2, 5, 10, 11].

The treatment of EM depends on the severity of the disease manifestation, the causes, and its acute or chronic course.

In most cases, EM is minor and regresses spontaneously in 2 to 4 weeks. Therapy is similar for EM minor and major with the addition of oral care for oral EM. When the manifestations are not severe, only symptomatic, conservative care is usually indicated. This may include topical analgesics and oral care which consist mainly of soothing mouth rinses, like viscous lidocaine rinse; topical anesthetics, such as gel benzocaine in Orabase Bland or lidocaine gel; soft liquid diet; and avoidance of spicy and acidic food. Adequate nutrition with high-calorie and high-protein diet is essential.

Systemic treatment may also be used such as systemic analgesics, as well as antibiotic treatment if the lesions are secondarily infected.

Some authors believe that the use of systemic glucocorticoids is unnecessary [10] in this case, and it may worsen the condition [8]. Contrary to acute EM, treatment with systemic corticosteroids and prednisone has been recommended [12], although controlled studies are missing.

In cases with a high suspicion of drug-induced EM, the first measure is to stop the drug or exposure to drugs with a potential for crossreactivity due to similar chemical structures [2].

In patients with recurrent episodes, especially with a history of HSV infection, antiviral therapy is recommended and may be beneficial in preventing recurrences. Tatnall et al. performed a 6-month double-blind placebo-controlled study with 20 patients with recurrent EM, whereof 15 had a proven HSV association, with acyclovir 400 mg twice daily [13]. The study showed significant superiority of acyclovir compared to placebo treatment with regard to the prevention of further EM episodes. Furthermore, after discontinuation of acyclovir, a fraction of patients remained in clinical remission, whereas all patients treated with placebo showed recurrence [2].

In patients with HSV-associated EM resistant to antiviral therapy, oral cyclosporine, thalidomide with its known TNF-*α* modulating mode of action despite its teratogenic effect [2], and corticosteroid-sparing drugs, such as dapsone, azathioprine, methotrexate, and mycophenolate mofetil, may also be considered for these challenging patients [5].

## 4. Conclusion

Due to its typical clinical and histological features, its frequent association with HSV, and its potentially recurrent course, EM represents a distinct entity [2]. As there remain no specific diagnostic tests, early clinical recognition of disease remains essential to promptly initiate appropriate treatment [14] and improve life quality.

Thus, in the above case, prompt diagnosis and immediate treatment given to the patient not only cured him but also prevented any recurrence, thereby saving the patient from major discomfort and pain [15].
